# Risk factors for poor outcomes of spontaneous supratentorial cerebral hemorrhage after surgery

**DOI:** 10.1007/s00415-021-10888-w

**Published:** 2021-11-17

**Authors:** Kangwei Zhang, Lai Wei, Xiang Zhou, Baoqing Yang, Jinxi Meng, Peijun Wang

**Affiliations:** 1 Department of Radiology, School of Medicine, Tongji Hospital, Tongji University, Shanghai, 200065 China; 2 Department of Radiology, Shuguang Hospital, Shanghai University of Traditional Chinese Medicine, Shanghai, 201203 China

**Keywords:** Cerebral hemorrhage, Predictive factors, Surgical procedures, Prognosis

## Abstract

**Objective:**

This study aimed to explore the factors affecting the outcomes of spontaneous supratentorial cerebral hemorrhage 90 days after surgery.

**Methods:**

A total of 256 patients with spontaneous supratentorial intracerebral hemorrhage underwent craniotomy evacuation of hematoma. The control group included 120 patients who received conservative treatment. The patients were divided into two subgroups based on a bifurcation of the modified Rankin Scale (mRS) 90 days after clinical therapeutics: good outcome (mRS score 0–3) and poor outcome (mRS score 4–6). The differences in clinical and imaging data between the two subgroups were analyzed. Based on difference analysis results, a binary logistic regression model was constructed to analyze the influencing factors related to poor outcomes.

**Results:**

The difference analysis results in the surgery group showed statistically significant differences in age, sex, Glasgow Coma Score (GCS) on admission, coronary atherosclerosis, smoking, stroke history, blood glucose, D-dimer, hematoma size, deep cerebral hemorrhage, midline shift, hematoma burst into the ventricle, vortex sign, island sign, and black hole sign. Binary logistic regression analysis showed that deep cerebral hemorrhage, midline shift, and age > 58 years independently correlated with the poor outcomes of patients after surgery. The binary logistic regression results of the control group showed that age > 58 years and GCS ≤ 8 independently correlated with the poor outcomes of patients.

**Conclusions:**

Deep cerebral hemorrhage, midline shift, and age > 58 years significantly increased the risk of adverse prognosis in patients after surgery. The findings might help select the clinical treatment plan and evaluate the postoperative prognosis of patients.

## Introduction

Spontaneous intracerebral hemorrhage (sICH) refers to spontaneous and non-traumatic cerebral vascular rupture. It characterized by high morbidity, mortality, and disability rates. At present, the surgical treatment of sICH mainly includes craniotomy evacuation of hematoma, minimally invasive surgery, and decompressive craniectomy. Craniotomy evacuation of hematoma can quickly and effectively remove the intracranial hematoma and reduce the mass effect and the cytotoxic effect of blood substances, so as to reduce intracranial pressure and prevent the occurrence of cerebral herniation, which is an important means to save the life of patients [1]. Patients with cerebellar hemorrhage who are deteriorating neurologically or who have brainstem compression and/or hydrocephalus from ventricular obstruction should get the hemorrhage surgically removed as soon as possible [2–4].

Two large clinical randomized controlled trials of supratentorial cerebral hemorrhage (STICH and STICH II) [5, 6] showed no significant gains from early surgery over conservative medical treatment. Patients with superficial lobar ICH without intraventricular hemorrhage might have a potential survival benefit. Current relevant studies and guidelines pointed out a high crossover rate between the conservative treatment group and the surgical treatment group in the aforementioned two experiments due to severe coma or cerebral herniation, which reduced mortality in the conservative treatment group. Additionally, comatose patients and patients at risk of cerebral herniation were not included. In these cases, surgery might be lifesaving [1]. To sum up, although definitive evidence favoring surgical intervention is lacking, a good theoretical rationale exists for early surgical intervention. Patients with intracerebral hemorrhage have the potential to benefit from surgery, which needs more individual analysis. The ideal patients who would benefit from early surgery are still to be determined.

Many factors influence the postoperative clinical outcomes of ICH. The factors significantly related to the poor outcomes of patients with ICH were summarized, providing a reference for the clinical choice of surgery, assisting the clinical evaluation of postoperative outcomes of patients, and hopefully improving the overall clinical outcomes of patients with ICH. At present, most of the relevant studies focus on the entire population of patients with intracerebral hemorrhage (whether to receive surgical treatment or not), and research on the risk factors of the patient population after the craniotomy evacuation of hematoma is lacking.

In this study, we retrospectively analyzed the relationship between the outcomes of 256 patients with ICH who underwent craniotomy evacuation of hematoma 90 days after surgery and the clinical and imaging indicators. The independent risk factors for poor outcomes after surgery were determined to assist the clinical evaluation of postoperative outcomes and individualized treatment options.

## Methods

### Patients

A total of 256 patients with the spontaneous supratentorial intracerebral hemorrhage who underwent craniotomy in Shanghai Tongji Hospital consecutively from March 2012 to October 2020 were retrospectively examined. The control group included 120 patients with supratentorial intracerebral hemorrhage who received conservative treatment in Tongji Hospital consecutively from January 2020 to September 2020. Imaging examinations were completed in all patients within 24 h of onset. No such patient was excluded from consideration in the time frame of the study. To obtain a homogenous population, patients with traumatic intracerebral hemorrhage, epidural hemorrhage, spontaneous subarachnoid hemorrhage, aneurysm rupture, subtentorial intracerebral hemorrhage, and brainstem hemorrhage were excluded. The study was approved by the Institutional Review Board (approval number: K-2020-021), and informed consent was exempted due to the retrospective nature of the study. These procedures were carried out in accordance with relevant guidelines and regulations.

### Craniotomy evacuation of hematoma

All patients were treated according to the Chinese guidelines for diagnosis and treatment of acute intracerebral hemorrhage 2019 [4]. The medical conservative treatment was given when a patient was generally in good condition, had no progressive aggravation of neurological symptoms, did not meet the surgical treatment indication, or could not tolerate surgery due to old age. When the hematoma volume was larger than 30 mL, neurological symptoms were progressively aggravated or cerebral herniation occurred, and life was endangered. In such cases, craniotomy evacuation of hematoma should be considered. An assessment was made by responsible chief physicians or deputy chief physicians to determine whether the patient was suitable for surgery. All the surgeries were performed in Shanghai Tongji Hospital by the responsible chief physicians or deputy chief physicians. The patient received intravenous combined anesthesia, and a horseshoe incision was made on the scalp nearest to the hematoma. The dural membrane was radially opened. In principle, the important functional areas and vascular areas of the brain were avoided, the cortex was cut along the direction of the cerebral gyrus until the hematoma area, and the hematoma was cleared under the microscope. After the hematoma was removed, hemostasis was completely stopped, an epidural tube was placed for drainage, and routine cranial closure was performed. Decompressive craniectomy was performed in patients with severe brain swelling after hematoma clearance. The drainage tube was usually removed 3–7 days after the surgery. If the hematoma burst into the ventricle, one or both ventricles were drained at the same time.

### End-point

According to the modified Rankin Scale (mRS) 90 days after surgery, the patients were divided into two groups: the good-outcome group with an mRS score of 0–3, and the poor-outcome group with an mRS score of 4–6. In our study, the patients who met the criteria for craniotomy were generally in serious condition, and the proportion of the patients whose mRS score was in the range of 4–6 was large. Therefore, we defined the result of mRS dichotomy as 0–3/4–6, rather than 0–2/3–6[5, 7–9]. The 90-day mRS data were collected mainly through telephone interviews and outpatient and clinical medical records. In telephone interviews, patients were asked about their functional recovery 90 days after treatment (including whether they were able to walk independently and needed help with daily activities), which assessed the degree of disability or dependence in daily activities, with scores ranging from 0 (no symptoms) to 6 (death).

### Acquisition of clinical, radiological, and biological data

We retrospectively collected 25 parameters by analyzing each patient’s characteristics on inclusion. The clinical information, including age, sex, smoking, drinking, diabetes, coronary atherosclerosis, hypertension, stroke history, time from onset to operation (not included in the conservative treatment group), systolic blood pressure on admission, diastolic blood pressure on admission, GCS score on admission, and hematoma clearance rate (not included in the conservative treatment group), was routinely collected. The biological parameters at baseline, including blood glucose, C-reactive protein (CRP), D-dimer, and plasma brain natriuretic peptide (BNP), were also analyzed. In terms of imaging parameters, we focused on hematoma size, deep cerebral hemorrhage, midline shift, hematoma burst into the ventricle, vortex sign, mixed sign, island sign, and black hole sign. Deep intracerebral hemorrhage refers to intracerebral hemorrhage involving the thalamus, basal ganglia region, and internal capsule, and can also extend to the superficial cerebral lobe. The midline shift was measured at the cerebral falx, septum pellucida, and pineal gland. They represented the upper, middle, and lower layers of the supratentorial brain. When more than two parts were displaced at the same time, the part with the largest displacement distance was recorded. The criterion of the midline shift was that the structures of the cerebral falx, pineal gland, and septum pellucida deviated more than 4 mm from the vertical line of the attachment point before and after the falx cerebellum. The size of hematoma was calculated using the Coniglobus formula (A × B × C/2), and the hematoma clearance rate = hematoma clearance volume/hematoma volume × 100%. To ensure the quality of imaging assessment, blinded independent analyses were retrospectively performed by two neuro-radiologists, and a chief radiologist made the judgment in the case of disagreement.

### Imaging signs

**Fig. 1:**
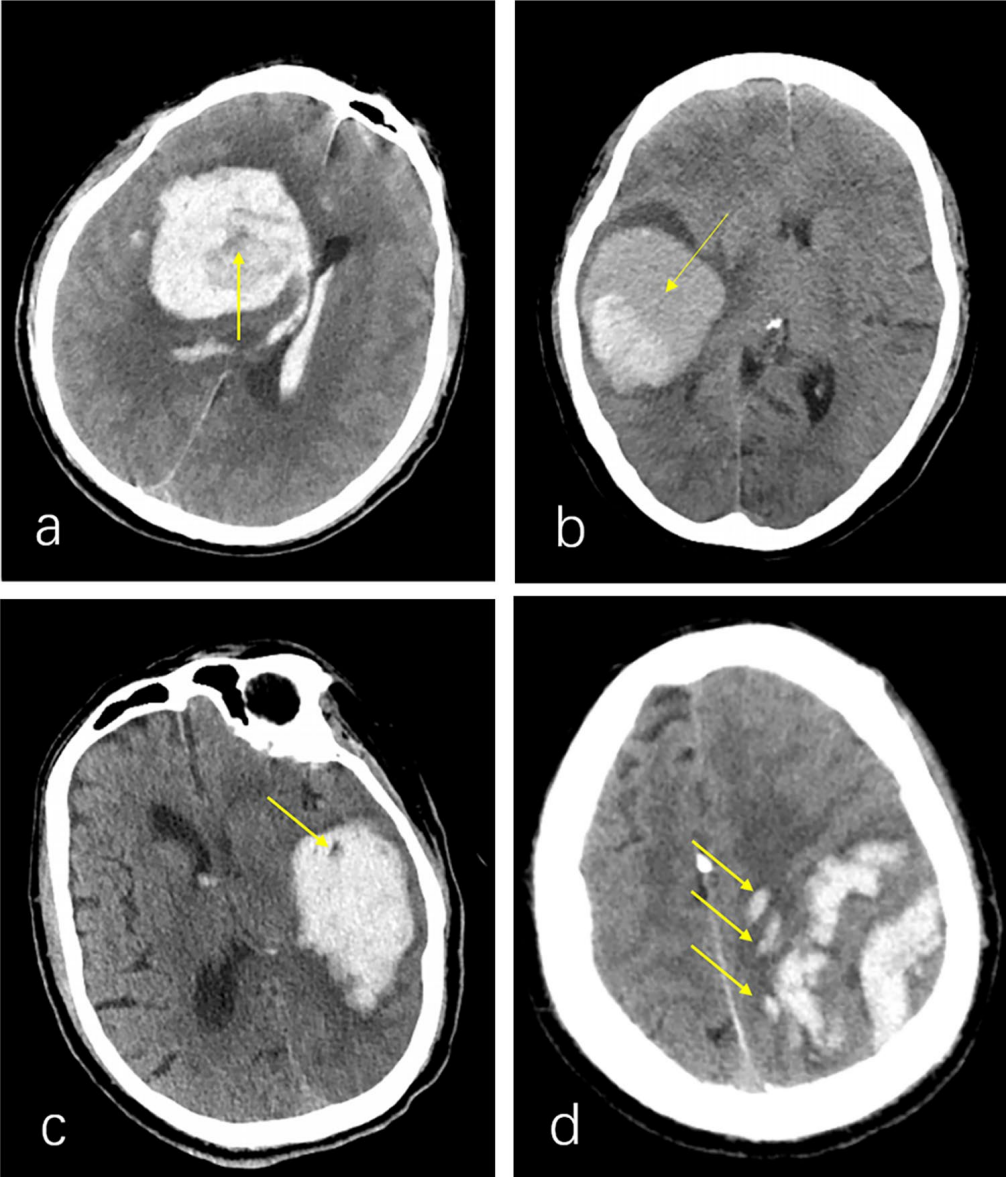
Examples of vortex signs (**a**), mixed signs (**b**), black hole signs (**c**), and island signs (**d**)

As shown in Fig. 1. Black hole sign: it is a well-defined, low-density area in a high-density hematoma that is not connected to the surrounding brain tissue and may be circular, oval, or bar-shaped, with a difference of at least 28 HU from the computed tomography (CT) value of the surrounding hematoma [10]. Island sign: the number of small hematomas surrounding the large hematoma should be more than 3; if all or part of the hematoma is connected, the number should be more than 4 [11]. Vortex sign: it is a low-density or iso-density area within a high-density area of the hematoma. The shape may be round, striped, or irregular [12]. Mixed sign: it includes both relatively high and low-density areas in the hematoma; the boundary between them is clear, with a CT value difference of more than 18 HU [13].

### Statistical analysis

SPSS 20.0 statistical software package was used for data processing. Continuous variables were expressed as means [± standard deviation (SD)] or medians [± interquartile range (IQR)] and categorical variables as numbers (percentage, range 0–100%). The normality of distributions was assessed using histograms and the Shapiro–Wilk test. Normally distributed variables were presented as means (± SD). Continuous variables between groups were compared using the Mann–Whitney *U* test, whereas binary categorical data between the groups were compared using the Pearson Chi-square test. Significant variables (*P* < 0.05) were selected for further multivariate analysis, a binary logistic regression model was constructed, and the correlation between multiple variables and clinical outcomes was explored. The model variables were tested for multicollinearity, and the variables with multicollinearity were excluded. By applying Bonferroni correction to the statistical significance threshold, the cutoff for the *P* value was equal to 0.05/*n* (where *n* is the number of independent variables being evaluated), minimizing errors due to the multiplicity of comparisons. The effects of the logistic regression model fits were evaluated using the Hosmer–Lemesow goodness-of-fit test.

## Results

### Demography data of patients

In this study, the information of 376 patients in Shanghai Tongji Hospital was collected, and ICH was diagnosed by emergency head CT. A total of 353 patients were finally enrolled due to the lack of follow-up information of some cases. Tables 1, 2, 3 compare the baseline characteristics of the surgery and control groups. A higher percentage of patients in the surgery group had poor outcomes compared with those in the control group, but with no significant difference. In terms of sex distribution, the two groups were essentially the same. The proportion of male patients was higher (164, 70.4%). The average age of patients in the control group (68.14 ± 13.78) was higher than that in the surgery group (58.66 ± 13.68); it was possible that most patients treated conservatively were too old to tolerate surgery. About two-thirds of the patients in the surgery group (155, 66.5%) were comatose (GCS ≤ 8), which was significantly more than that in the control group (30, 25%). The volume of hematoma in the surgery group (median, 101.38; IQR, 67.65–155.41) was significantly larger than that in the control group (median, 27.27; IQR, 10.30–50.93). The blood pressure in both groups increased significantly at admission, and the diastolic pressure in the surgery group (median 106; IQR, 90–120) increased more significantly than that in the control group (median 100; IQR, 89–113). The number of patients with a history of atherosclerosis in the control group (56, 46.7%) was significantly higher than that in the surgery group (22, 9.4%), which might be related to the different age distribution of patients in the two groups. As the symptoms of patients in the surgery group were relatively severe, the blood glucose level would increase significantly under stress; the blood glucose level in the surgery group (median 8.15; IQR, 6.80–9.97) was significantly higher than that in the control group (median, 7.24; IQR, 6.01–8.76). Among the imaging features, the proportion of hematoma burst into the ventricle, vortex sign, mixed sign, and island sign in the surgery group was significantly higher than that in the control group. In the surgery group, the time from onset to surgery was mostly within 12 h (172, 73.8%). The mean hematoma clearance rate was 60% (median 58.0%; IQR 41.0%–83.0%). The distribution of other measures did not differ significantly between the two groups. Most patients had no history of smoking (35, 15.0%), drinking (32, 13.7%), diabetes (34, 14.6%), and stroke (27, 11.6%). Further, 95 (40.8%) patients had hematoma bursting into the ventricle. Most patients had intracerebral hemorrhage in deep functional areas (179, 76.8%). The probability of the midline shift was 34.8% in the surgery group and 27.5% in the control group. Of the three midline transpositions we measured, septum pellucidum transposition accounted for the largest proportion [in surgery group, 68 (84.0%); in control group, 18 (54.5%). Also, 42 patients in the surgical group had black hole signs on non-contrast CT images. The d-dimer level (median, 1.11 mg/L; IQR, 0.3–2.3 mg/L) in most patients was higher than normal levels. The plasma BNP (median, 74.2 pg/mL; IQR, 37.7–155.0 pg/mL) and CRP levels (median, 2.77 mg/L; IQR 1.02–8.01 mg/L) were mostly in the normal range.

**Table 1:** Baseline clinical characteristics of patients in the two groups

Variable	Surgery (*n* = 233)	Conservative treatment (*n* = 120)	*P* value
mRS 90 days after surgery			0.063
Good outcome (%)	83 (35.6)	55 (45.8)	
Poor outcome (%)	150 (64.4)	65 (54.2)	
Age, year	58.66 ± 13.68	68.14 ± 13.78	< 0.001
Age, year			0.003
> 58 (%)	129 (55.4)	87 (72.5)	
≤ 58 (%)	104 (44.6)	33 (27.5)	
GCS score on admission			< 0.001
> 8 (%)	78 (33.5)	90 (75)	
≤ 8 (%)	155 (66.5)	30 (25)	
Systolic blood pressure on admission, mm Hg	180.90 ± 29.05	176.23 ± 27.70	0.095
Diastolic blood pressure on admission, mm Hg	106.00 (90.00–120.00)	100.00 (89.00–113.00)	0.017
Hematoma clearance rate (%)	58.0 (41.0–83.0)	–	–
Sex			0.940
Male (%)	164 (70.4)	84 (70)	
Female (%)	69 (29.6)	36 (30)	
Time from onset to operation, h		–	–
< 12 (%)	172 (73.8)	–	–
≥ 12 (%)	61 (26.2)	–	–
Smoking (%)	35 (15.0)	26 (21.7)	0.118
Drinking (%)	32 (13.7)	20 (16.7)	0.461
Diabetes (%)	34 (14.6)	27 (22.5)	0.063
Coronary atherosclerosis (%)	22 (9.4)	56 (46.7)	< 0.001
Hypertension (%)	193 (82.8)	101 (84.2)	0.750
Stroke history (%)	27 (11.6)	22 (18.3)	0.083

**Table 2:** Preoperative biological information of patients in the two groups

Variable	Surgery (*n* = 233)	Conservative treatment (*n* = 120)	*P* value
Blood glucose, mmol/L	8.15 (6.80–9.97)	7.24 (6.01–8.76)	< 0.001
CRP, mg/L	2.77 (1.02–8.01)	2.46 (0.87–6.52)	0.235
d-dimer, mg/L	1.11 (0.33–2.35)	0.70 (0.28–1.87)	0.077
BNP, pg/mL	74.20 (37.70–155.00)	60.70 (34.93–130.14)	0.274

**Table 3:** Preoperative imaging characteristics of patients in the two groups

Variable	Surgery (*n* = 233)	Conservative treatment (*n* = 120)	*P* value
Deep cerebral hemorrhage (%)	179 (76.8)	89 (74.2)	0.580
Hematoma size, mL	101.38 (67.65–155.41)	27.27 (10.30–50.93)	< 0.001
Midline shift (%)	81 (34.8)	33 (27.5)	0.167
Cerebral falx	4 (4.9)	4 (12.1)	
Septum pellucida	68 (84.0)	18 (54.5)	
Pineal gland	9 (11.1)	11(33.3)	
Hematoma burst into the ventricle (%)	95 (40.8)	33 (27.5)	0.014
Vortex sign (%)	139 (59.7)	39 (32.5)	< 0.001
Mixed sign (%)	89 (38.2)	19 (15.8)	< 0.001
Island sign (%)	141 (60.5)	21 (17.5)	< 0.001
Black hole sign (%)	42 (18.0)	14 (11.7)	0.121

### Pre‑procedural predictors of clinical outcomes

Univariate analyses for potential predictors of clinical outcomes are reported in Tables 4, 5, 6. Among the baseline clinical characteristics in the surgery group, age > 58 years, admission GCS score ≤ 8 points, smoking, coronary atherosclerosis, and stroke history increased the risk of poor outcomes. In the conservative treatment group, age > 58 years, admission GCS score ≤ 8 points, systolic blood pressure on admission, coronary atherosclerosis, and stroke history increased the risk of poor outcomes. The analysis of sex variables in the surgery group showed that although the prevalence of cerebral hemorrhage in women was lower than that in men, the risk of poor outcomes for women was higher than that for men, as in the control group. This difference was not statistically significant in the subgroup analysis of the control group. Among biological characteristics, elevated blood glucose and d-dimer levels increased the risk of poor outcomes in the surgery group. A subgroup analysis of the conservatively treated group showed that only the elevated d-dimer level was associated with poor outcomes. Among the imaging features, large hematoma size, midline shift, hematoma burst into the ventricle, vortex sign, island sign, and black hole sign were associated with poor outcomes, in both the surgery group and the conservative treatment group. Moreover, deep cerebral hemorrhage was also associated with poor outcomes in the surgery group.

**Table 4:** Univariate analysis of clinical baseline characteristics in the two groups

Variable	Surgery		*χ*^2^/*Z*	*P* value	Conservative treatment		*χ*^2^/*Z*	*P* value
	Good outcome (*n* = 83)	Poor outcome (*n* = 150)			Good outcome (*n* = 55)	Poor outcome (*n* = 65)		
Age, year	52.06 ± 11.33	62.31 ± 13.53			61.60 ± 11.31	73.68 ± 13.31		
Age, year			30.15	< 0.001			10.44	≤ 0.001
> 58 (%)	26 (31.3)	103 (68.7)			23 (41.8)	10 (15.4)		
≤ 58 (%)	57 (68.7)	47 (31.3)			32 (58.2)	55 (84.6)		
GCS score on admission			8.77	0.003			20.69	< 0.001
> 8 (%)	38 (45.8)	40 (26.7)			52 (94.5)	38 (58.5)		
≤ 8 (%)	45 (54.2)	110 (73.3)			3 (5.5)	27 (41.5)		
Systolic blood pressure on admission, mm Hg	183.82 ± 30.29	179.29 ± 28.31	5812.50	0.402	167.95 ± 24.06	183.25 ± 28.80	1233.50	0.003
Diastolic blood pressure on admission, mm Hg	110.00 (96.00–120.00)	104.00 (90.0–119.25)	5359.50	0.078	100.00 (89.00–113.00)	100.00 (89.00–113.00)	1784.00	0.985
Hematoma clearance rate (%)	64.0 (41.0–85.0)	57.0 (41.0–81.0)	5734.00	0.319	–	–	–	–
Sex			5.16	0.023			3.24	0.072
Male (%)	66 (79.5)	98 (65.3)			43 (78.2)	41 (63.1)		
Female (%)	17 (20.5)	52 (34.7)			12 (21.8)	24 (36.9)		
Time from onset to surgery, h			0.05	0.820	–	–	–	–
< 12 (%)	62 (74.7)	110 (73.3)			–	–	–	–
≥ 12 (%)	21 (25.3)	40 (26.7)			–	–	–	–
Smoking (%)	19 (22.9)	16 (10.7)	6.26	0.012	14 (25.5)	12 (18.5)	0.86	0.354
Drinking (%)	15 (18.1)	17 (11.3)	2.05	0.152	7 (12.7)	13 (20.0)	1.14	0.287
Diabetes (%)	9 (10.8)	25 (16.7)	1.45	0.228	11(20.0)	16 (24.6)	0.36	0.546
Coronary atherosclerosis (%)	2 (2.4)	20 (13.3)	7.46	0.006	19 (34.5)	37 (56.9)	5.99	0.014
Hypertension (%)	72 (86.7)	121 (80.7)	1.39	0.239	48 (87.3)	53 (81.5)	0.74	0.391
Stroke history (%)	3 (3.6)	24 (16.0)	8.00	0.005	23 (41.8)	14 (21.5)	5.75	0.017

**Table 5:** Univariate analysis of preoperative biological information in the two groups

Variable	Surgery		*χ*^2^/*Z*	*P* value	Conservative treatment		χ^2^/Z	*P* value
	Good outcome (*n* = 83)	Poor outcome (*n* = 150)			Good outcome (*n* = 55)	Poor outcome (*n* = 65)		
Blood glucose, mmol/L	7.50 (6.39–8.81)	8.76 (7.09–10.36)	4505.00	< 0.001	7.05 (5.88–8.64)	7.36 (6.21–9.44)	1510.00	0.144
CRP, mg/L	2.82 (1.11–8.00)	2.64 (0.94–8.03)	6159.50	0.894	2.41 (0.41–4.90)	2.82 (1.14–10.84)	1483.50	0.109
D-dimer, mg/L	0.68 (0.26–2.35)	1.62 (0.35–2.74)	5043.00	0.016	0.38 (0.24–1.87)	0.94 (0.38–2.08)	1356.00	0.023
BNP, pg/mL	76.60 (34.20–155.00)	74.10 (39.58–155.00)	6035.50	0.700	55.7 (27.80–124.10)	68.50 (36.75–172.30)	1432.00	0.061

**Table 6:** Univariate analysis of imaging characteristics in the two groups

Variable	Surgery		*χ*^2^/*Z*	*P* value	Conservative treatment		*χ*^2^/*Z*	*P* value
	Good outcome (*n* = 83)	Poor outcome (*n* = 150)			Good outcome (*n* = 83)	Poor outcome (*n* = 150)		
Deep cerebral hemorrhage (%)	55 (66.3)	124 (82.7)	8.04	0.004	38 (69.1)	51 (78.5)	1.37	0.243
Hematoma size, mL	90.31 (64.02–120.59)	113.31 (70.47–171.22)	4738.00	0.003	–	–	–	–
Midline shift (%)	15 (18.1)	66 (44.0)	15.84	< 0.001	7 (12.7)	26 (40.0)	11.11	≤ 0.001
Hematoma burst into the ventricle (%)	26 (31.3)	69 (46.0)	4.77	0.029	8 (14.5)	25 (38.5)	8.55	0.003
Vortex sign (%)	42 (50.6)	97 (64.7)	4.392	0.036	10 (18.2)	29 (44.6)	9.49	0.002
Mixed sign (%)	34 (41.0)	55 (36.7)	0.418	0.518	7 (12.7)	12 (18.5)	0.74	0.391
Island sign (%)	38 (45.8)	103 (68.7)	11.710	≤ 0.001	4 (7.3)	17 (26.2)	7.36	0.007
Black hole sign (%)	7 (8.4)	35 (23.3)	8.028	0.005	2 (3.6)	12 (18.5)	6.35	0.012

Variables with *P* < 0.05 after univariate analysis were included in our multivariate analysis model. Multicollinearity diagnosis was carried out before constructing the binary logistic regression model. The variance inflation factor (VIF) of all the selected variables were less than 10, and multicollinearity did not exist. The dependent variable of the logistic regression model was the two-category mRS results 90 days after treatment: 0–3/4–6 points. After the Bonferroni correction of 15 independent variables in the surgery group and 11 independent variables in the control group, the threshold of statistical significance was defined as *P* = 0.003 in the surgery group and *P* = 0.005 in the control group. Table 7 lists all the variables included in the final logistic regression model of the surgery and control groups. In the surgery group, deep cerebral hemorrhage [odds ratio (OR), 4.068; 95% CI, 1.835–9.021; *P* ≤ 0.001], midline shift (OR, 3.989; 95% CI, 1.902–8.368; *P* < 0.001), and age (OR, 6.308; 95% CI, 3.108–12.800; *P* < 0.001) were independent predictors of clinical outcomes 90 days after surgery. In the control group, age (OR, 6.70; 95% CI, 2.14–20.99; *P* ≤ 0.001) and GCS score on admission (OR, 16.78; 95% CI, 3.85–73.09; *P* < 0.001) were independent predictors of clinical outcomes 90 days after conservative treatment. The Hosmer–Lemesshow goodness-of-fit test showed no statistically significant difference between the predicted value of the regression model and the observed value, indicating that the model fit well in both the surgery (*P* = 0.988) and control groups (*P* = 0.942).

**Table 7:** Binary logistic regression analysis

Variable	Surgery			Conservative treatment		
	OR	95% CI	*P* Value	OR	95% CI	*P* Value
Deep cerebral hemorrhage	4.07	1.84–9.02	≤ 0.001	Not included	Not included	Not included
Midline shift	3.99	1.90–8.37	< 0.001	Not included	Not included	Not included
Age	6.31	3.11–12.80	< 0.001	6.70	2.14–20.99	≤ 0.001
Island sign	2.41	1.24–4.65	0.009	Not included	Not included	Not included
Stroke history	5.05	1.30–19.62	0.019	Not included	Not included	Not included
Vortex sign	Not included	Not included	Not included	2.86	1.02–7.97	0.045
Systolic blood pressure on admission	Not included	Not included	Not included	1.02	1.00–1.04	0.017
GCS score on admission	Not included	Not included	Not included	16.78	3.85–73.09	< 0.001

## Discussion

**Fig. 2:**
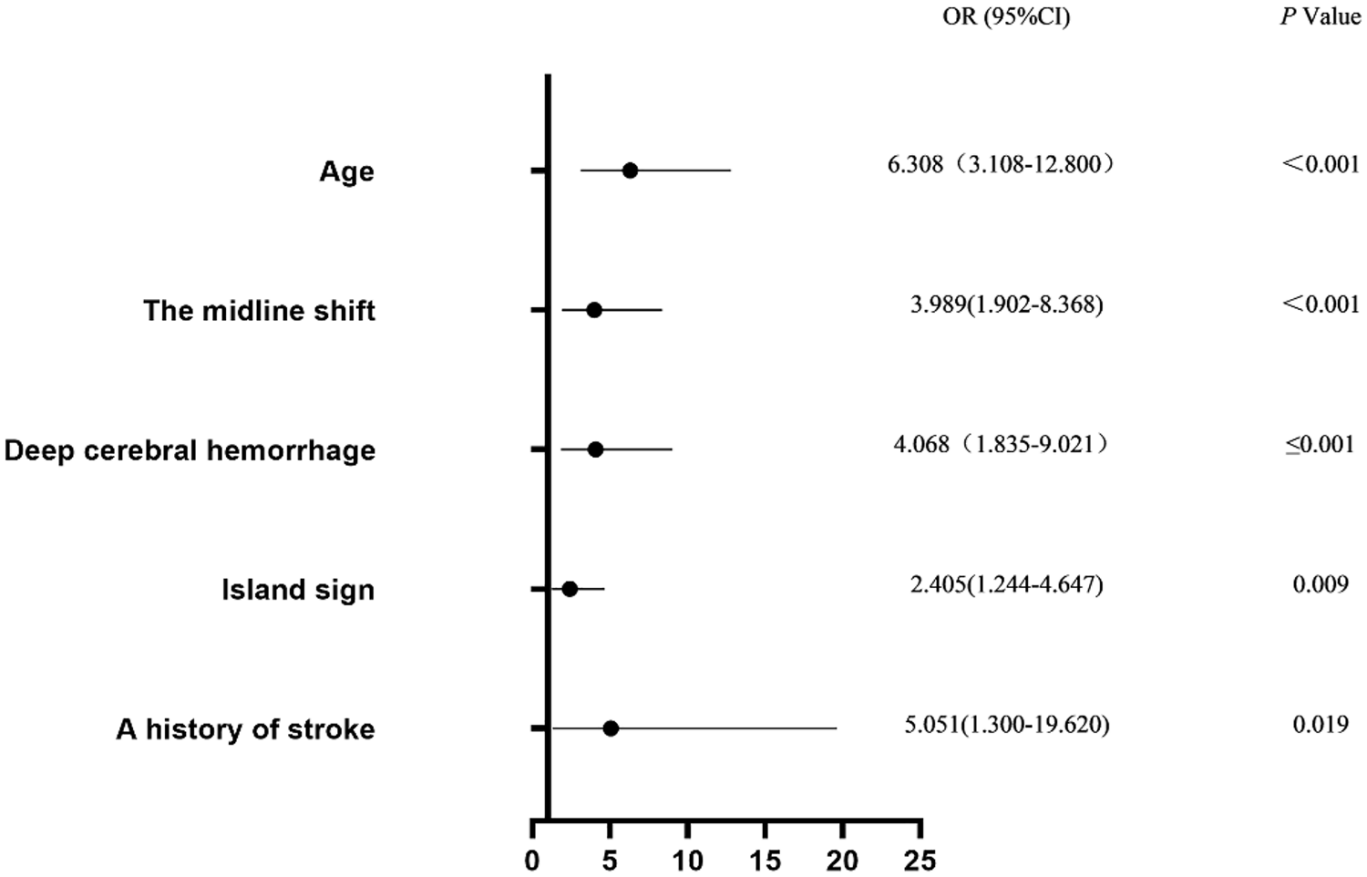
Forest plot of binary logistic regression results of the surgery group

**Fig. 3:**
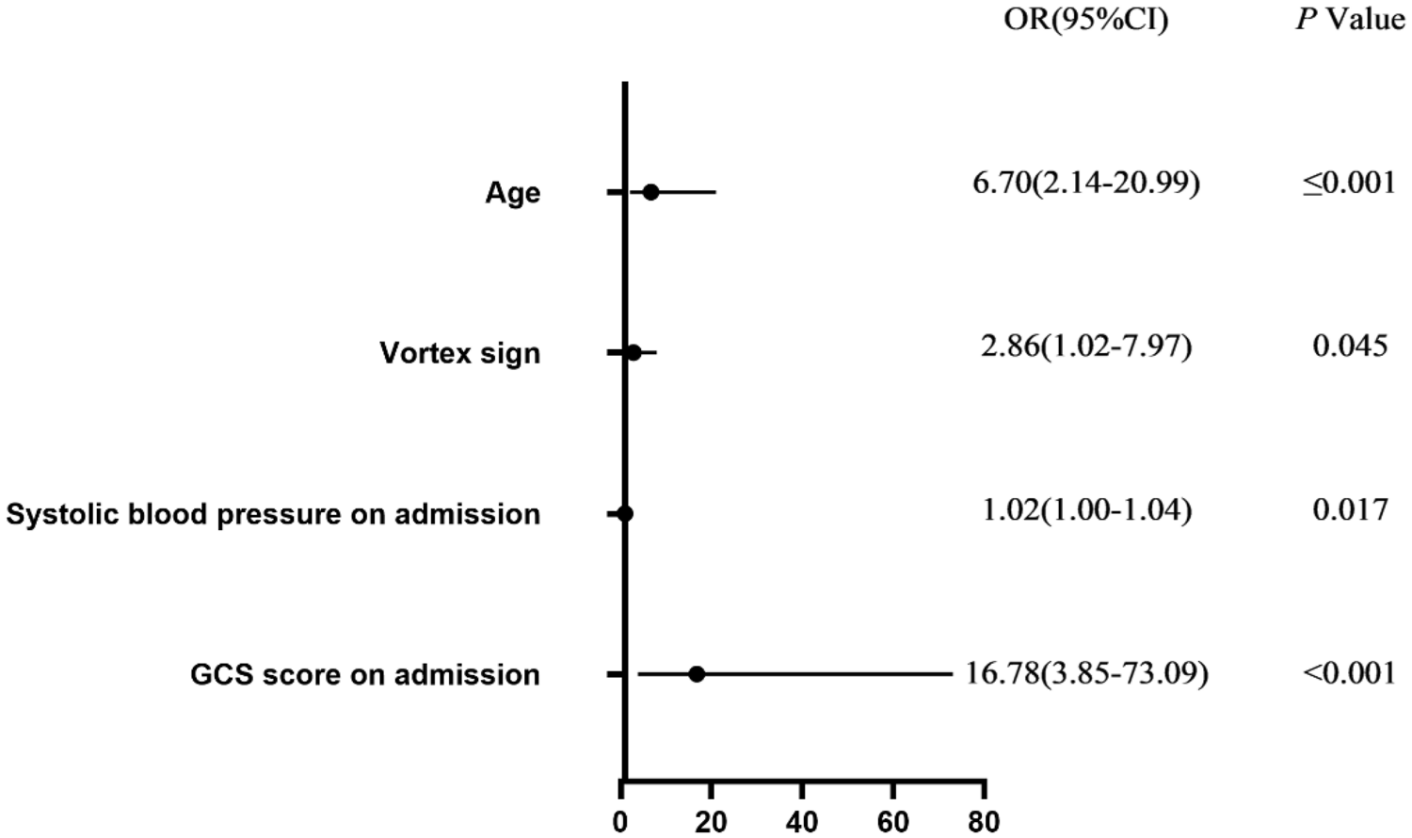
Forest plot of binary logistic regression results of the control group

Figures 2 and 3 show the relationship between variables in the logistic regression model and poor outcomes. In the surgery group, patients with deep intracerebral hemorrhage, midline shift, and age > 58 years were at varying risks for poor outcomes. In contrast, patients with age > 58 years and GCS score on admission ≤ 8 were at varying risks for poor outcomes in the control group.

Previous studies confirmed that the involvement of deep functional areas in cerebral hemorrhage was closely related to the poor outcomes of patients, which might be caused by the destruction of fiber bundles in functional areas, resulting in corresponding clinical symptoms. In studies on ischemic stroke, diffusion tensor fiber-tract imaging showed that internal capsule posterior limb injury was associated with poor motor function outcomes and disability [14]. Thalamic hemorrhage has a higher in-hospital mortality rate than intracerebral hemorrhage at other supratentorial locations [15]. Hemorrhage from the thalamus was more likely to infiltrate the ventricles and extend to compress the brain stem with life-threatening consequences [16]. Delcourt et al. confirmed that ICH involving the thalamus and posterior limbs of the internal capsule was associated with death (OR 1.72), major disability (OR 2.26), and health index scale (EQ-5D) scores (OR 1.71) [17]. In this study, the proportion of deep cerebral hemorrhage in the poor-outcome group (124, 82.7%) was greater than that in the good-outcome group (55, 66.3%). The logistic regression analysis showed that deep cerebral hemorrhage was an independent risk factor for poor postoperative outcomes (OR, 4.07; 95% CI, 1.84–9.03; *P* ≤ 0.001).

Midline shift is caused by hematoma compression, which can cause neuronal damage in the early stage of intracerebral hemorrhage and is associated with poor outcomes. Previous studies showed that early evacuation of the mass effect of intracerebral hemorrhage and active clinical intervention effectively improved the prognosis of patients [18]. This seems to contradict our findings, which might be due to the fact that in our study, the time from onset to surgery was more than 6 h (median 6.70; IQR, 4.33–13.00). At this time, the brain damage caused by the mass effect had already occurred and was irreversible. Studies suggested that the impact of midline shifts in different parts on the prognosis of patients with intracerebral hemorrhage was different, septum pellucidum shift was a sensitive sign of mass effect after intracerebral hemorrhage, and the displacement of the pineal gland was associated with the level of consciousness and mortality [19–21]. This might be due to the injury of different functional areas in the midline, and the corresponding neurological dysfunction, which affected the prognosis of patients. In our study, a midline shift occurred in 81 (34.8%) patients in the surgery group, Among the three sites we measured, the septum pellucida accounted for the largest 68 (84.0%). On the one hand, it might be because the septum pellucidum was sensitive to mass effect; on the other hand, it might be because spontaneous intracerebral hemorrhage was more likely to occur in the basal ganglia and thalamus, which were located in the middle layer of the supratentorial brain. Yang et al. argued that the maximum midline shift > 4 mm was the best threshold for poor prognosis in patients with supratentorial ICH within 6 h after onset [22]. Hallevy et al. showed that the midline shift was strongly associated with decreased levels of consciousness and poor outcomes (OR, 19.78; 95% CI, 4.61–84.77; *P* < 0.001) [23]. The results of our study were the same as previous findings. The risk of poor outcomes significantly increased when patients with intracerebral hemorrhage had a midline shift (OR, 3.99; 95% CI, 1.91–8.37; *P* < 0.001).

It is commonly known that age is an independent risk factor for poor outcomes in patients with intracerebral hemorrhage. Aging is usually accompanied by some basic diseases, such as diabetes, cardiovascular disease, and history of antithrombotic therapy, which are associated with a poor prognosis of intracerebral hemorrhage to varying degrees [24, 25]. Health-related quality of life after stroke was worse with age [26, 27]. Almost half of people aged more than 75 years had anxiety or depression. Previous studies showed that older patients with stroke were more likely to suffer from depression [28, 29]. Depressive symptoms and reduced social support led to reduced health outcomes even after treatment for physical function and severe stroke [27]. In Rådholm et al. study, advanced age was strongly associated with the severity of intracerebral hemorrhage and poor outcomes, including death or severe disability after 90 days [30]. In our study, patients with poor outcomes (62.3 ± 13.5) were significantly older than those with good outcomes (52.1 ± 11.3), in both the surgery and control groups. Patients aged > 58 years had a 6.31-fold increased risk of poor postoperative outcomes compared with patients aged ≤ 58 years (OR, 6.31; 95% CI, 3.11–12.80; *P* < 0.001).

Troberg et al. retrospectively analyzed clinical and radiographic information from 229 patients with intracerebral hemorrhage who underwent the craniotomy evacuation of hematoma. Using case fatality as an end-point event, the final results showed that heart disease and level of consciousness were independent predictors of mortality [31]. Heart disease includes a wide range of diseases that this study did not define. Coronary atherosclerosis was included as an independent variable in our study, and the univariate analysis showed a statistically significant difference between the two groups (*P* = 0.006). The difference between our study and the study by Troberg et al. was that patients with subtentorial cerebral hemorrhage were excluded from our study. Consciousness disturbances of cerebellar and brainstem hemorrhage are usually more serious than supratentorial cerebral hemorrhage. Compared with our study, the study by Troberg et al. showed that the level of the conscious state of patients might have a stronger correlation with the final clinical outcome. In our study, 155 patients (100%) had a GCS of ≤ 8 in the surgery group, and 45 (29%) patients had a good outcome. In the control group, 30 (100%) patients had a GCS of ≤ 8, and 3 (10%) patients had a good prognosis. The difference between the two groups was statistically significant (*P* < 0.001), which suggested that surgery improved the clinical outcome of comatose patients in our study cohort. In our study, the subgroup analysis of the surgery group showed that a GCS score of ≤ 8 was associated with poor outcomes in patients with spontaneous supratentorial hemorrhage (*P* < 0.001). A GCS score of ≤ 8 in the control group was included as an independent adverse prognostic factor in the final logistic regression model (*P* < 0.001). Also, different from Troberg et al. ’s study, the prognostic follow-up time in our study was uniform, 90 days after surgery. The difference in the follow-up time of the prognosis of intracerebral hemorrhage might result in a large difference in the outcome, which might also be one of the factors leading to different results in the final study. In our study, deep cerebral hemorrhage, midline shift, and age > 58 years were independent risk factors for poor outcomes in patients with cerebral hemorrhage after surgery. Comparative literature is lacking due to the lack of relevant studies on the prognostic factors of patients with intracerebral hemorrhage who underwent craniotomy evacuation of hematoma.

This study had some limitations. First, this was a single-center study, and the difference in the surgical level in different hospitals might have had a certain impact on the prognosis of patients. Second, this was a retrospective study, and the results were susceptible to selection bias. Finally, the sample size of this study was small and should be further increased in future studies.

## Conclusions

Deep cerebral hemorrhage, midline shift, and age > 58 years significantly increased the risk of adverse prognosis in patients with spontaneous supratentorial intracerebral hemorrhage after surgery. The finding might provide some help for the selection of clinical treatment plan and the postoperative prognostic evaluation of patients with spontaneous supratentorial intracerebral hemorrhage.
